# Retinal Perfusion Analysis of Children with Diabetes Mellitus Type 1 Using Optical Coherence Tomography Angiography

**DOI:** 10.3390/jpm14070696

**Published:** 2024-06-28

**Authors:** Jelena Vasilijevic, Igor Kovacevic, Snezana Polovina, Bojana Dacic-Krnjaja, Tanja Kalezic, Suzana Miletic, Leila Al Barri, Simona Stanca, Francis Ferrari, Maja Jesic

**Affiliations:** 1 Faculty of Medicine, University of Belgrade, 11000 Belgrade, Serbia; dr.igor.kovacevic@gmail.com (I.K.); bkrnjaja@gmail.com (B.D.-K.); tanjakalezic@gmail.com (T.K.); maja.jesic@udk.bg.ac.rs (M.J.); 2 Clinic for Eye Diseases, Clinical Centre of Serbia, 11000 Belgrade, Serbia; 3 Clinic for Endocrinology, University Clinical Centre of Serbia, 11000 Belgrade, Serbia; snezanapolovina@gmail.com; 4 Pediatric Clinic, Clinical Centre of Kosovska Mitrovica, 38220 Kosovska Mitrovica, Serbia; suzanadrmiletic@gmail.com; 5 Department of Ophthalmology, “Victor Babes” University of Medicine and Pharmacy, 300041 Timisoara, Romania; leila.albarri@umft.ro; 6 Department of Pediatrics, “Carol Davila” University of Medicine and Pharmacy, 050474 Bucharest, Romania; simona.stanca@umfcd.ro; 7 Centre d’Ophtalmologie FUTURA, 67300 Strasbourg, France; francisferrari@me.com; 8 Department of Pediatric Endocrinology, University Children’s Hospital, 11000 Belgrade, Serbia

**Keywords:** retinal perfusion, optical coherence tomography angiography, type 1 diabetes, vessel density, treatments

## Abstract

(1) Background: This study aims to evaluate retinal perfusion by optical coherence tomography angiography (OCTA) in pediatric patients with type 1 diabetes mellitus (T1D) without diabetic retinopathy (DR). (2) Methods: Thirty-one patients affected by T1D were enrolled. All participants were evaluated using OCTA. The foveal avascular zone (FAZ) and superficial and deep macular vessel density (VD) were analyzed. The correlation of these parameters with metabolic factors such as body mass index (BMI), glycated hemoglobin (HbA1c), and the type of insulin therapy (multiple daily injections, MDI vs. continuous subcutaneous insulin infusion, CSII) was determined. (3) Results: None of the OCTA parameters were significantly different between the groups. The patients’ HbA1C level did not influence any of the OCTA parameters. The use of MDI tended to reduce the parafoveal and perifoveal deep VD (*p* = 0.048 and *p* = 0.021, respectively) compared to CSII. An elevated BMI tended to increase the deep macular (*p* = 0.005) and perifoveal VD (*p* = 0.006). (4) Conclusion: VD and FAZ are normal in pubescent children with T1D without signs of DR. Treatment with CSII may be a better choice compared to MDI, as CSII may be protective against retinal microvascular damage. Our results indicate the need for new clinical parameters of glycemic control in addition to HbA1c which could assess the risk of DR.

## 1. Introduction

Diabetes is a group of metabolic diseases characterized by an increase in glucose levels as a result of either a defect in insulin secretion or an increase in resistance to its activity [1]. Among children and adolescents, diabetes is the third most common chronic disease, with autoimmune type 1 diabetes (T1D) being the most common. Early pediatric age onset with suboptimal metabolic control causes failure and damage to different organs such as the eyes, the kidneys, nerves, or the heart [1,2]. Diabetic retinopathy (DR) is one of the most common complications of diabetes, which leads to vision impairment and blindness if not treated in time. The occurrence of DR is rare before puberty, especially in children under the age of 10, so the early detection of DR through screening programs is crucial for preserving vision in patients with diabetes [3,4]. Indirect ophthalmoscopy and invasive fluorescein angiography are diagnostic methods commonly used worldwide and provide the highest accuracy in detecting DR [4,5]. With the introduction of optical coherence tomography angiography (OCTA), a new diagnostic tool, quantitative assessment of the microcirculation in the macula and optic nerve head has been made possible. OCTA has improved our understanding of glaucoma and various retinal diseases [6,7,8,9], including DR. Due to its non-invasiveness, it is very suitable for children.

To the best of our knowledge, there are very few articles on the role of OCTA in the preclinical diagnosis of DR in pediatric patients [2,4,10], and these presented conflicting results. Therefore, in this study, we aimed to evaluate retinal perfusion via OCTA in pediatric patients with T1D without clinically evident DR and to compare these findings with a group of age- and sex-matched healthy controls. 

## 2. Materials and Methods

### 2.1. Study Design and Population 

This research was conducted at the Clinic for Eye Diseases, University Clinical Center of Serbia, Belgrade. It was a cross-sectional study. Thirty-one consecutive patients affected by T1D who were followed up as per routine clinical care were enrolled in this study. The inclusion criteria were diagnosis of T1D without clinically evident DR and age ≤ 19 years. Control subjects were defined as having a normal ophthalmic examination and no history of diabetes. Exclusion criteria were history of eye disease that significantly reduces visual acuity, larger refractive errors (greater than ±6D spheres and/or greater than ±3D cylinder), history of retinal vascular disease or the presence of maculopathy due to other diseases, previous significant ocular trauma, and intraocular pressure greater than 21 mmHg. This study is part of a subspeciality work, which was approved by the Ethics Committee of the Faculty of Medicine, University of Belgrade, record number 22-UEK-11, on 20 September 2023. Informed consent was obtained from all patients participating in the study (>16 years old) or from the parents (of those patients ≤ 16 years old) after receiving a full explanation of the study.

### 2.2. Clinical and Ophthalmological Assessment

The following data were collected for each T1D patient: gender, age, body mass index (BMI), disease duration, systolic and diastolic blood pressure, glycated hemoglobin (HbA1c) levels, total cholesterol, low-density lipoprotein cholesterol (LDL), triglycerides, and type of insulin therapy (multiple daily injections, MDI, vs. continuous subcutaneous insulin infusion, CSII).

All patients and control participants underwent a complete ophthalmological examination (biomicroscopy evaluation of the anterior segment, measurement of the IOP by Goldmann applanation tonometry, and fundus examination by indirect ophthalmoscopy). After that, OCTA was performed with an Optovue, Fremont, CA, USA, device. The central macular thickness (CMT) within 1 mm of the central retina was registered, expressed in microns. The OCTA measurements we monitored were the foveolar avascular zone (FAZ) in mm^2^, the density of the vascular network (vessel density—VD) (expressed in %) of the superficial and deep capillary plexus (SCP, DCP), and the flow area of the outer retina (OR) and choriocapillaris (CC), expressed in mm^2^. The primary outcome measures were vascular density in the macular, foveal, parafoveal, and perifoveal regions in the superficial and deep layers, as well as flow area in the outer retina and choriocapillaris.

### 2.3. Statistical Analysis

Continuous variables were expressed as the mean and standard deviation; categorical data were expressed as numbers and percentages. Categorical variables were compared using the Chi-square test. We conducted the Shapiro–Wilk normality test to test the normality of the data. Comparisons of continuous variables between groups were performed using the independent-samples *t*-test. The Spearman correlation coefficient was used to analyze the correlation between the variables. All statistical analyses were performed using SPSS for Windows, version 20.0 (SPSS Inc., Chicago, IL, USA). A value of *p* < 0.05 was considered statistically significant.

## 3. Results

### 3.1. Characteristics of the Participants

A total of 41 participants were included in this study. Of these, 31 patients (75.6%) were in the T1D group, while 10 participants (24.4%) were in the control group. The mean of the patients’ ages was 14.68 ± 2.30 years (range, 8–19 years), and in the control group, it was 13.6 ± 3.44 years (range, 10–18 years). There were 14 females (45.16) and 17 males (54.84%) in the T1D group, while the control group was equally men and women. No statistically significant difference was determined between the groups with respect to age and gender (*p* = 0.935, *p* = 0.790, respectively). The average duration of T1D was 7.16 ± 3.59 years. The fundus examinations were normal without signs of diabetic retinopathy, while the visual acuity was 20/20 for all patients. The characteristics of all patients are summarized in Table 1.

### 3.2. Central Macular Thickness, Vessel Density, FAZ, and Flow Area Parameters of OCTA

The mean ± SD CMT was 248.8 ± 17.6 μm in the T1D group and 250.0 ± 18.3 μm in the control group. The difference between the two study groups was not statistically significant (*p* = 0.858). None of the monitored OCTA parameters were statistically significantly different between the groups, as shown in Table 2. Among the patients with T1D, we assessed correlations regarding the investigated OCTA parameters and potential predictors, i.e., diabetes duration (years), level of glycated hemoglobin (%), BMI, and type of T1D treatment. The diabetes duration and HbA1C levels did not significantly affect any of the OCTA parameters. Yet, treatment with MDI tended to reduce the parafoveal and perifoveal deep vessel density (ρ = 0.364, *p* = 0.048 and ρ = 0.420, *p* = 0.021, respectively). On the other hand, the use of CSII reduced the flow area in the OR (ρ = −0.372, *p* = 0.043). In addition, an elevated BMI tended to increase the deep macular (ρ = 0.502, *p* = 0.005) and deep perifoveal vessel density (ρ = 0.490, *p* = 0.006). The correlations between the diabetes duration (years), level of glycated hemoglobin (%), BMI, and type of T1D treatment and all the OCTA parameters are shown in Table 3.

## 4. Discussion

Pediatric patients are a significant and sensitive population for T1D. In the last 20–30 years, there has been a noted decreasing incidence of DR in children with diabetes [11], most likely as a result of more effective treatment, the use of insulin pumps, and better education of children and their families. Due to its long duration, T1D affects all organs, including the eyes [2]. In our study, no child had clinical signs of diabetic retinopathy upon fundus examination, in terms of the presence of microaneurysms as an early form of DR. According to some studies, preclinical DR may be accompanied by early vascular abnormalities of the capillaries before the appearance of microaneurysms [2]. We performed OCTA to analyze potential structural changes in retinal and choroidal microvasculature before the appearance of clinical signs of DR and to correlate them with clinical parameters in a pediatric population. 

Although some previous OCTA-based studies have reported several retinal microvasculature abnormalities in pediatric diabetic patients without DR [2], in our study, none of the monitored OCTA parameters were statistically significantly different between the T1D and control groups. Our results are consistent with Gołębiewska et al., who found normal vessel density, both in superficial and deep plexuses, and FAZ area in pubescent children with T1D compared to healthy subjects [4]. Kim Duong et al. also found no significant changes in the FAZ and vascular density in their T1D cohort of <15 years using OCTA [10]. On the other hand, the authors suggested that in their cohort, changes in the density of retinal vessels occurred very early, before the appearance of other diabetes-related complications, and they pointed out that the retina is one of the most sensitive target tissues [2].

Our study monitored the correlations between diabetes duration, the level of glycated hemoglobin, BMI, and the type of T1D treatment with OCTA parameters. The patients’ diabetes duration and HbA1C level did not significantly affect any OCTA parameters. Several studies have indicated a lack of correlation between HbA1c level and retinal vessel density in patients with T1D [2,10], which is consistent with the results of our study. Maneli et al. postulate that the lack of correlation with HbA1c means that perhaps we should look for new metrics in clinical practice to assess the risk of chronic complications associated with diabetes [2]. Yet, the authors found in their study that an elevated level of HbA1C was associated with reduced parafovea superficial vessel density in pubescent children with T1D [4]. Due to these disagreements in the literature, some authors suggest greater attention to new clinical parameters, such as the so-called “time in range”, which is described as the percentage of time a patient spends within their target glucose range, whose value has already been confirmed in type 2 diabetes [12,13]. “Time in range” is derived from continuous glucose monitoring and provides information on whether the frequency and duration of glycemic fluctuations improve over time, and it is useful for evaluating different glycemic-lowering treatments [13].

In our study, an elevated BMI tended to increase the deep macular and perifoveal vessel density. By searching the literature, we found results that indicate that an increase in BMI is associated with an increase in VD in the superficial and deep layers, but in adults [14]. Zhang et al., in their study of healthy children aged 8–16 years, did not find that BMI affected OCTA parameters such as vascular density and FAZ [15]. On the other hand, Han et al. found higher VD in the fovea of SCP and DCP in children with newly developed obesity [16]. They explained that endothelial dysfunction and oxygen deficiency in obese children caused a reduction in local capillaries, which led to the compensatory dilation of surrounding retinal capillaries. Can et al. speculate in their study that the increased VD found in children with obesity might be associated with too many proinflammatory cytokines [17].

When we analyzed the difference between the groups that received different treatment methods, we found a significantly lower parafoveal and perifoveal deep vessel density in the multiple daily insulin injections group. The obtained results are not surprising, considering that in children on continuous subcutaneous insulin infusion as a more physiological form of treatment, we expect better retinal perfusion. In their study, Guo et al. suggested that a CSII may be a better choice for children with T1DM to prevent retinal complications from DR, considering the significantly lower VD values in children who received multiple daily insulin injections compared to CSII, with similar HbA1c [18]. Downie et al. found that the use of CSII was independently associated with lower rates of DR and better metabolic control among young T1D patients [11]. This information could be very useful for young T1D patients and their parents. Unfortunately, there are still insufficient numbers of patients receiving CSII for both diabetes types.

Some potential limitations of this study can be noted. In a cross-sectional design, the association between two variables should be interpreted carefully, and this study could only assess the status of the retinal vasculature at a specific point in time. Secondly, when interpreting these results, the size of the study sample definitely should be taken into account, as the small sample size limits the power of this study and the ability to detect small but potentially clinically significant differences. Future longitudinal studies could be needed to address these issues and to investigate the use of OCTA parameters as potential biomarkers in predicting the course of DR.

## 5. Conclusions

In summary, we provided preliminary insights that treatment with CSII may be a better choice to prevent retinal complications than MDI, as it provides greater protection against retinal microvascular damage in the stages before the onset of DR. Also, our results could indicate the need for novel clinical parameters of glycemic control besides HbA1c which could assess the risk of DR. On the other hand, vessel density and the FAZ area are normal in pubertal children with T1D without signs of DR. The role of OCTA in patients with T1DM remains to be determined in future longitudinal studies.

## Figures and Tables

**Table 1:** The clinical characteristics of patients with T1D.

Investigated Trait	Mean	Standard Deviation (±SD)
Age (years)	14.68	2.30
Diabetes duration (years)	7.16	3.59
Glycated hemoglobin (%)	8.83	1.64
Cholesterol level (mmol/L)	4.40	0.94
Triglyceride level (mmol/L)	0.95	0.58
LDL level (mmol/L)	2.26	0.69
Body mass index (BMI)	21.14	2.98
Systolic blood pressure (mmHg)	108.33	10.03
Diastolic blood pressure (mmHg)	69.67	8.30
Treatment type; *n* (%)		
Multiple daily insulin injections (MDI)	19 (63.3%)	
Continuous subcutaneous insulin infusion (CSII)	11 (36.7%)	

T1D—type 1 diabetes; LDL—low-density lipoprotein; BMI—body mass index; MDI—multiple daily insulin injections; CSII—continuous subcutaneous insulin infusion.

**Table 2:** Comparison between groups as regards the optical coherence tomography angiography outcomes.

	Group	T1D	Control	*p*-Value
OCTA Parameters				
Superficial macula density (%)		48.38 ± 3.28	49.47 ± 2.22	0.333
Superficial foveal density (%)		21.77 ± 6.07	18.29 ± 6.95	0.136
Superficial parafoveal density (%)		51.57 ± 4.20	52.85 ± 2.60	0.372
Superficial perifoveal density (%)		48.90 ± 3.48	49.53 ± 2.47	0.600
Deep macula density (%)		47.62 ± 4.76	49.35 ± 3.42	0.294
Deep foveal density (%)		37.76 ± 8.20	38.81 ± 5.09	0.705
Deep parafoveal density (%)		54.05 ± 3.70	53.62 ± 5.03	0.728
Deep perifoveal density (%)		50.59 ± 5.53	50.22 ± 5.43	0.343
Flow rate OR		0.71 ± 0.39	0.72 ± 0.46	0.376
Flow rate CC		2.20 ± 0.08	2.22 ± 0.12	0.504
FAZ area (mm^2^)		0.22 ± 0.10	0.26 ± 0.05	0.257

OR: outer retina; CC: choriocapillaris; FAZ: foveal avascular zone; T1D: type 1 diabetes; Values are stated as mean ± standard deviation.

**Table 3:** The correlation between the diabetes duration (years), level of glycated hemoglobin (%), BMI, and type of T1D treatment and all OCTA parameters.

	Diabetes Duration	Glycated Hemoglobin	BMI	Type of T1D Treatment
Superficial macula density				
CC	0.028	−0.125	0.166	0.140
Sig.	0.883	0.517	0.380	0.467
Superficial foveal density				
CC	−0.083	−0.216	0.115	−0.085
Sig.	0.665	0.261	0.546	0.661
Superficial parafoveal density				
CC	−0.089	−0.156	0.207	0.132
Sig.	0.638	0.420	0.272	0.496
Superficial perifoveal density				
CC	−0.020	−0.100	0.202	0.132
Sig.	0.917	0.605	0.286	0.495
Deep macula density				
CC	−0.064	−0.108	0.502 **	0.288
Sig.	0.732	0.571	0.005	0.123
Deep foveal density				
CC	−0.016	−0.020	0.257	−0.260
Sig.	0.930	0.918	0.171	0.166
Deep parafoveal density				
CC	0.115	−0.122	0.330	0.364 *
Sig.	0.538	0.519	0.075	0.048
Deep perifoveal density				
CC	0.085	0.002	0.490 **	0.420 *
Sig.	0.650	0.990	0.006	0.021
Flow rate OR				
CC	0.041	−0.016	−0.257	−0.372 *
Sig.	0.827	0.932	0.170	0.043
Flow rate CC				
CC	−0.140	0.032	−0.339	−0.052
Sig.	0.454	0.868	0.067	0.785
FAZ area				
CC	−0.095	0.023	−0.004	0.112
Sig.	0.611	0.905	0.984	0.556
CMT				
CC	0.240	−0.121	0.104	−0.268
Sig.	0.193	0.524	0.586	0.152

BMI—body mass index; OR—outer retina, CC—choriocapillaris; CMT—central macular thickness; T1D: type 1 diabetes; CC, correlation coefficient; Sig, significant; * Significant at the 0.05 level (two-tailed). ** Significant at the 0.01 level (two-tailed).

## Data Availability

The datasets used and/or analyzed during the current study are available without restriction from the corresponding author. All relevant data are contained within the paper.
